# TE-C36 carbon: a new semiconducting phase with an all-sp^3^ bonding network†

**DOI:** 10.1039/c7ra11448f

**Published:** 2018-01-08

**Authors:** Yanheng Xu, Yihua Lu, Xi Zhu, Min Wang

**Affiliations:** The Chinese University of Hong Kong No.2001 Longxiang Blvd., Longgang Dist. Shenzhen 518172 Guangdong China zhuxi@cuhk.edu.cn; Institute for Clean Energy and Advanced Materials, Faculty of Materials and Energy, Southwest University Chongqing 400715 China minwang@swu.edu.cn

## Abstract

A new carbon allotrope is investigated by first principles calculations. The allotrope consists of 36 atoms in a tetragonal cell and displays *P*4_2_/*nmc* symmetry (termed TE-C36 carbon) with a mass density of 3.18 g cm^−3^. The new carbon phase has an all-sp^3^ network, possessing squares, rhombuses, pentagons and hexagons formed by near-by atoms. The dynamic and mechanical stabilities are demonstrated by phonon dispersion and elastic constants, respectively. Its bulk modulus is 353 GPa. The analysis of its electronic band structure shows that it is a semiconductor possessing a direct band gap of 2.25 eV. X-ray diffraction patterns and Raman spectra are also simulated for future experimental characterization. Due to the direct band gap and a comparatively large bulk modulus, this new semiconducting carbon allotrope may possess not only potential electronic and optical applications but also mechanical application.

## Abstract 2

TE-C36 carbon, possessing an all-sp^3^ bonding network, is a semiconductor with a direct band gap of 2.25 eV.

## Graphical abstract

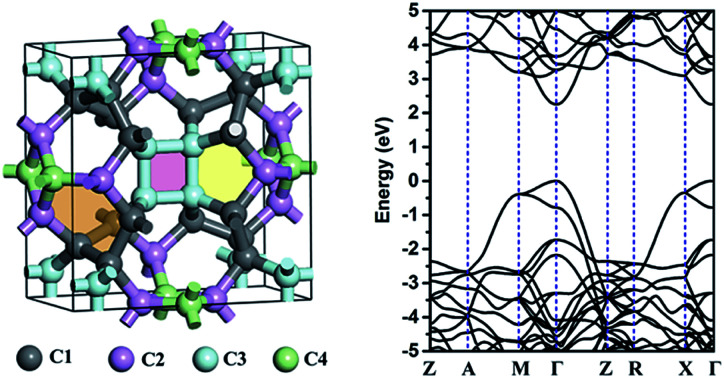

## Introduction

Carbon can form a variety of allotropes and organic compounds, due to its different hybridizations of sp, sp^2^ and sp^3^ when binding with other elements.^1^ Diamond and graphite are two natural carbon allotropes, and consist of sp^3^ and sp^2^ hybridization, respectively. Since the 1980s, the experimental discoveries of fullerene^2^ and carbon nanotubes^3^ have attracted great interest to the investigation of their syntheses, properties and applications. Since 2004, the observation of two-dimensional graphene^4^ has given rise to enormous novel scientific and technological exploration.^5–11^

Besides these familiar carbon allotropes, other carbon materials are also investigated. For example, a new carbon allotrope, obtained from the compression of graphite under ambient temperature,^12^ exhibits a superhard property, which is even harder than diamond. Motivated by the observation of the superhard allotrope, several carbon phases were theoretically proposed, including M carbon,^13^ W carbon^14^ and bct-C4 carbon.^15^ Additionally, new 2D carbon allotropes are proposed, such as penta-graphene,^16^ R-graphyne,^17^ net W carbon^18^ and net C carbon.^18^ Additionally, graphdiyne, one of the 2D carbon allotropes, has been synthesized experimentally, and it also shows excellent semiconducting property and possessed tunable band gap.^19,20^ Furthermore, sp-hybridized carbon allotropes are also predicted or synthesized, including one-dimensional sp-carbyne, two-dimensional sp-sp^2^-graphyne and three-dimensional sp-sp^3^-yne-diamond.^21,22^ Other carbon materials are also reported, such as K6 carbon,^23^ 3D3C carbon,^24^ C20-T carbon,^25^ CY carbon,^26^ C2/m-20 carbon^27^ and T-II carbon.^28^ Very recently, T carbon, which was theoretically predicted in 2011,^29^ has been experimentally observed.^30^ The grown T-carbon nanowire is obtained by the picosecond pulsed-laser irradiation of a multi-walled carbon nanotube suspension *via* pseudo-topotactic conversion.^30^ The successful synthesis of T carbon nanowire extends the field of carbon science, as it is the third identified 3D periodic carbon crystals except diamond and lonsdaleite, and most importantly, in a very recent work, the predicted T-carbon structure is successfully synthesised experimentally,^30^ which provides encouraging expectation for more carbon allotropes materials which are still in the modelling stages. Additionally, penta-graphene^16^ as a new 2D carbon allotrope is predicted by the exfoliation of T12-carbon,^31^ and the penta-graphene structure can form the 3D full pentagon carbon allotrope *via* the inter-layer interactions,^32^ by different packing of the pentagon unit, the full pentagon carbon allotrope indicates novel topological fermions characters.^33^ Inspired by recent progresses of new carbon allotropes, we investigate a new carbon allotrope to help further understand the carbon chemistry.

In this work, we theoretically investigate a new stable 3D semiconducting carbon allotrope with 36 atoms in a tetragonal cell in *P*4_2_/*nmc* symmetry (hereafter termed TE-C36 carbon). Different from the traditional diamond and graphite and the new synthesized T carbon, TE-C36 carbon is composed of square, rhombus, pentagon and hexagon, and it has a bulk modulus of 353 GPa and a mass density of 3.18 g cm^−3^. To our best knowledge, our new carbon allotrope is empty in the previous collected database.^34^

## Theoretical methods and models

Most theoretical calculations are performed by using density functional theory (DFT) based vienna *ab initio* simulation package (VASP) code^35^ by applying the projector augmented wave (PAW) method.^36^ Generalized gradient approximation (GGA) with Perdew–Burke–Ernzerhof (PBE) functional^37,38^ is adopted for the exchange and correlation potential. The energy cutoff is chosen as 500 eV. To approach full optimizations, the convergence for energy and force are set as 10^−4^ eV and 10^−3^ eV Å^−1^, respectively. Monkhorst–Pack *k*-point grid is set as 13 × 13 × 25 to sample Brillouin zone. The packages phonopy^39^ and vasp_raman.py^40,41^ are used for the calculations of phonon and Raman spectrum, respectively.

## Results and discussion

**Fig. 1:**
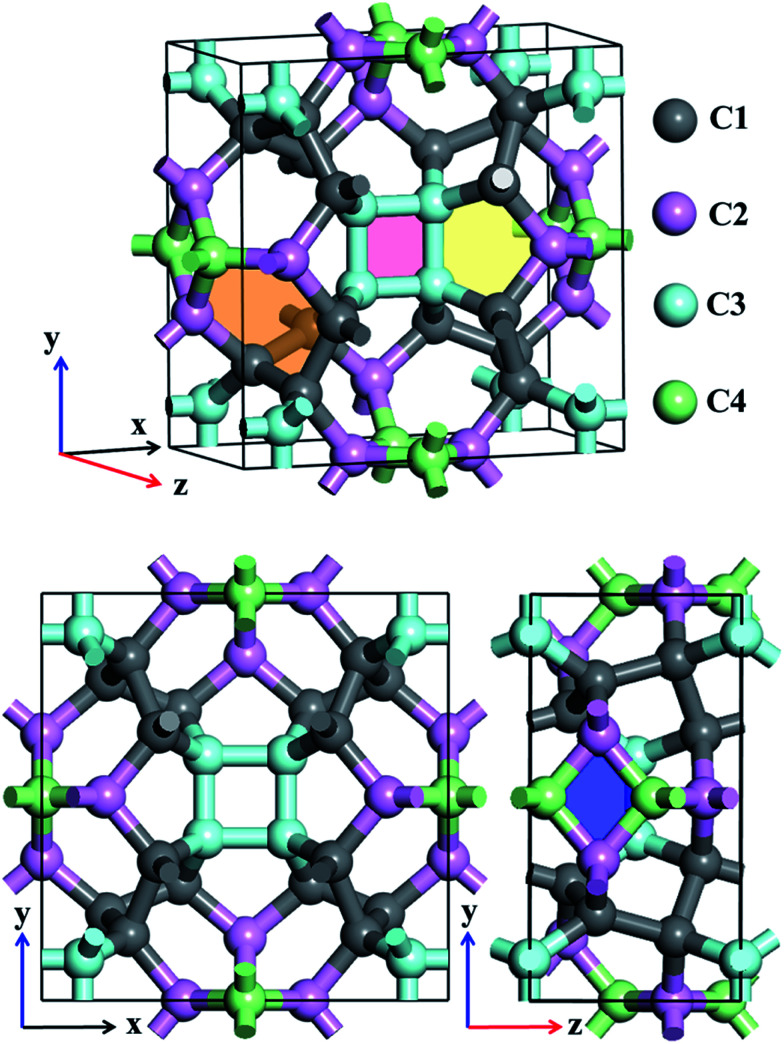
Schematic structure of TE-C36 carbon in a unit cell. Four inequivalent atoms are denoted as C1 (grey), C2 (light pink), C3 (light cyan) and C4 (light green). Light red, light blue, light yellow and light orange regions denote square, rhombus, pentagon and hexagon in CY carbon.

Atomic structures of TE-C36 carbon with the space group *P*4_2_/*nmc* (space group no. 137) is shown in Fig. 1 in the top- and side-view. The optimized lattice parameters are *a* = *b* = 7.583 Å, *c* = 3.931 Å, *α* = *β* = *γ* = 90°. Four inequivalent atoms occupy the crystallographic 16h (0.7872, 0.1595, 0.6893), 16h (0.8462, 0.5, 0.8246), 16h (0.6004, 0.6004, 0.5) and 16h (0, 0.5, 0.0734) positions in the unit cell, denoted as C1 (grey), C2 (light pink), C3 (light cyan) and C4 (light green) in Fig. 1.

Different from the traditional diamond and graphite, TE-C36 carbon is composed of square, rhombus, pentagon and hexagon, denoted by light red, light blue, light yellow and light orange regions in Fig. 1. The calculated bond lengths C1–C1, C1–C2, C1–C3, C2–C4, and C3–C3 are around 1.59 Å (or 1.56 Å), 1.58 Å, 1.56 Å, 1.52 Å (or 1.53 Å) and 1.52 Å, respectively. Note that the carbon bond lengths of graphite are 1.42 Å and those of diamond are 1.54 Å, revealing that the new phase possesses a sp^3^ networks. The bond angles C1–C1–C1, C1–C2–C1, C1–C1–C2, C1–C2–C3, C3–C2–C3, C2–C3–C2, C4–C4–C4, C1–C4–C4, C1–C1–C4 and C2–C1–C4 are 98.9° (or 75.5°), 100.1°, 117.0°, 117.8°, 80.2°, 99.5° (or 114.5°), 90°, 106.7°, 102.2° and 99.1°, respectively, revealing that the hybridization belonging to sp^3^ as well, in comparison to the bond angles (109.5° and 120°) of diamond and graphite for sp^3^ and sp^2^ hybridizations. We also notice that four C3 carbon atoms in a plane compose a square, denoted as a light pink region in Fig. 1. Additionally, two C2 and two C4 carbon atoms nearly in a plane compose a rhombus, denoted as a blue region in Fig. 1. The calculations also show that TE-C36 carbon has a density of 3.18 g cm^−3^, which is larger than the density of graphite (2.28 g cm^−3^) and smaller than the density of diamond (3.56 g cm^−3^).

**Fig. 2:**
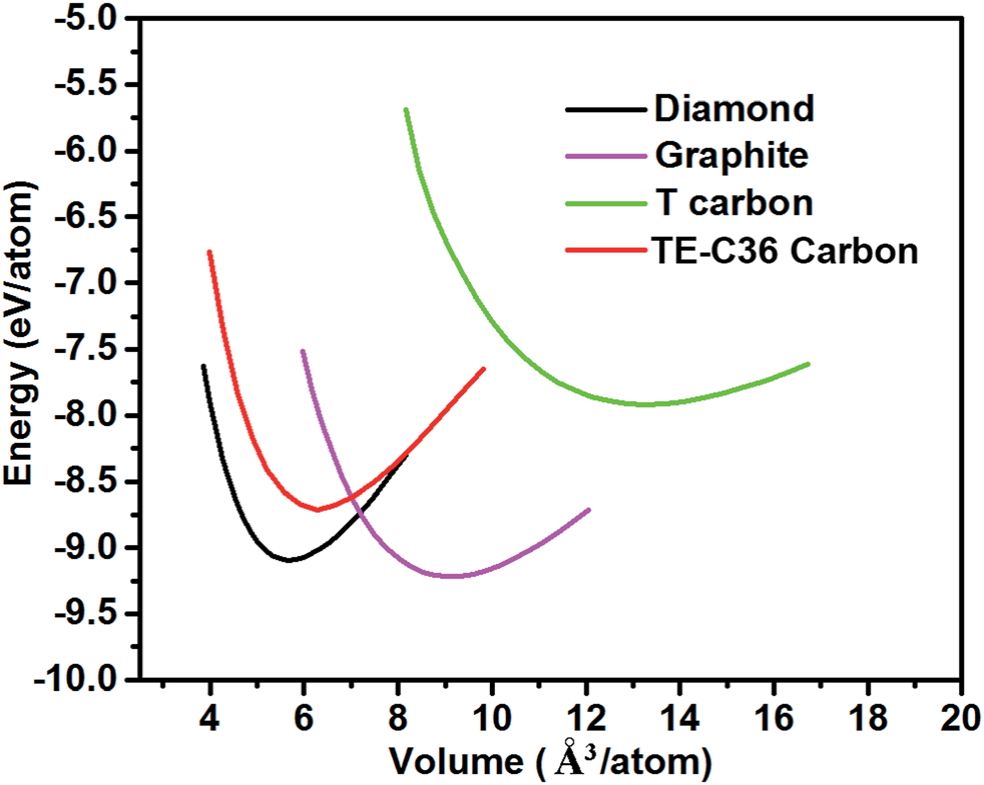
Total energies of different carbon allotropes as a function of volume (GGA-PBE calculations).

Fig. 2 plots the total energies of TE-C36 carbon as a function of volume, in comparison to diamond, graphite and T carbon. It is noted that TE-C36 carbon is less thermodynamically stable than graphite and diamond but is more stable than T carbon, thus it is metastable. The bond distortions in TE-C36 carbon contribute to the presence of strain, leading to the relative metastability.

The elastic constants matrix *C*_*ij*_ of TE-C36 carbon is calculated to estimate the mechanical stability. Due to the criteria of mechanical stability, the positive crystal deformation energy is needed, leading to the positive determinate of *C*_*ij*_.^42^ Due to the crystal symmetry, the elastic constants of tetragonal phase should satisfy the followings:^42^*C*_11_ > 0, *C*_33_ > 0, *C*_44_ > 0, *C*_66_ > 0, *C*_11_ − *C*_12_ > 0, *C*_11_ + *C*_33_ − 2*C*_13_ > 0, [2(*C*_11_ − *C*_12_) + *C*_33_ + 4*C*_13_] > 0.

It is noted that the calculated elastic constants matrix *C*_*ij*_ is
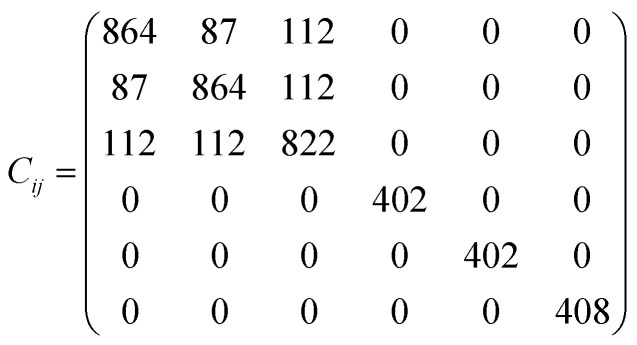
,so TE-C36 carbon satisfy the mechanical stability criteria, revealing that it is mechanically stable. Furthermore, with the Voigt–Reuss–Hill approximation,^43^ the estimated bulk modulus of TE-C36 carbon is 353 GPa, which is smaller than that of diamond (443 GPa)^44^ but is larger than T carbon (169 GPa).^29^

**Fig. 3:**
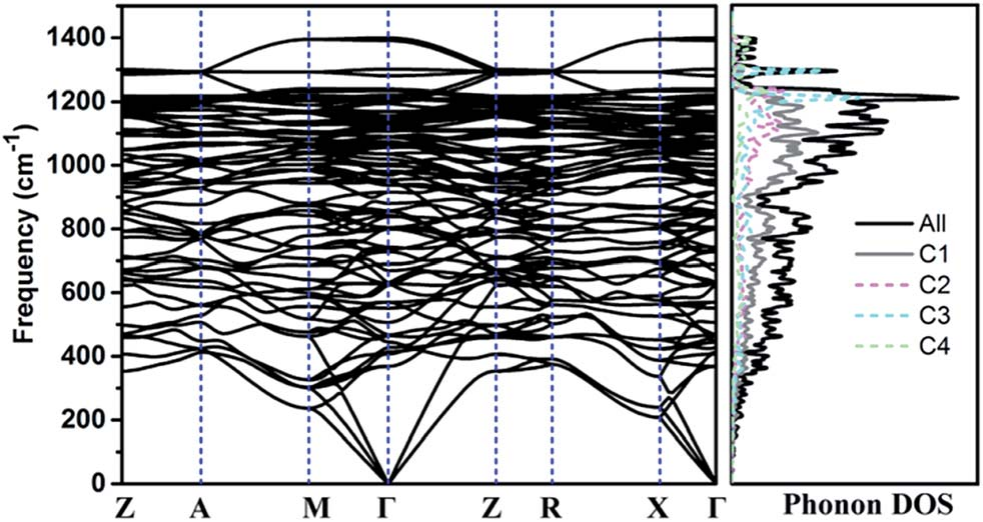
Phonon band structure and its density of states of TE-C36 carbon at 0 GPa. *Z*(0, 0, 0), *A*(0.5, 0.5, 0.5), *M*(0.5, 0.5, 0), *Γ*(0, 0, 0), *R*(0, 0.5, 0.5), *X*(0, 0.5, 0) are chosen for the *k*-path.

The calculated phonon band structure and phonon density of states are plotted in Fig. 3. No imaginary frequency at 0 GPa is observed through the whole Brillouin zone, implying that TE-C36 carbon is dynamically stable. The highest vibration frequency of TE-C36 carbon is around 1400 cm^−1^, which is close to ∼1400 cm^−1^ of sp^3^ bonded diamond, revealing that the frequency originates from sp^3^ bonds' vibration.^45^ The phonon PDOS curves demonstrated that the contributions of four inequivalent atoms are quite different. C1 atoms contribute the most when the frequency is below 1200 cm^−1^, while C3 atoms contribute the most when the frequency is larger than 1200 cm^−1^. These features of phonon dispersion and phonon PDOS can help to characterize the future experimental samples.

Since the data of temperature dependent phonon dispersion cannot be obtained by phonopy^39^ code, in order to further discuss the stability of this phase, we carried out *ab initio* MD simulations with the canonical (NVT) ensemble at 1000, 1500 and 2000 K, respectively. The system is modelling with 2 × 2 × 4 supercell. The time step is chosen 1 fs. The potential energy fluctuations of TE-C36 carbon in MD simulation at 1000, 1500 and 2000 K are presented in Fig. S1(a)–(c) (ESI†), respectively. It is noted that the potential energy fluctuations are quite small and TE-C36 carbon keeps its geometry after heating up to 1000 K for 50 ps. It is noted that though the potential energy fluctuations of the system have a little more variation at 1500 and 2000 K during the first 10 ps than those at 1000 K, the MD snapshots appearing at 5 ps, 25 ps and 50 ps, as shown in the inset of Fig. S1 (ESI†), demonstrate that the phase can still sustain its structure even at 2000 K.

**Fig. 4:**
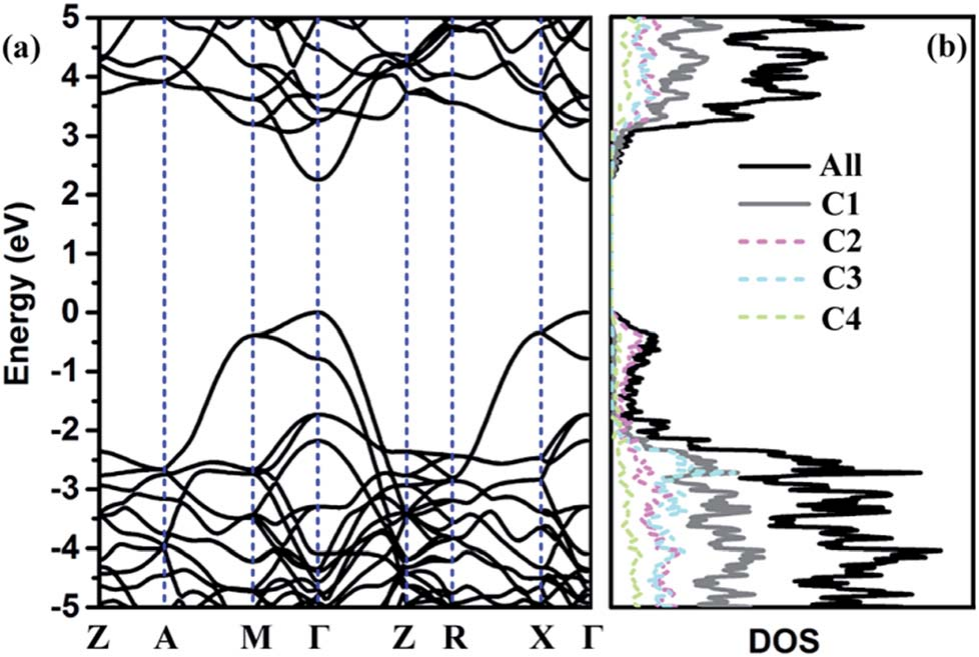
(a) Band structure and (b) partial density of states of C1, C2, C3 and C4 atoms and all atoms. Fermi level is set to zero. *Z*(0, 0, 0), *A*(0.5, 0.5, 0.5), *M*(0.5, 0.5, 0), *Γ*(0, 0, 0), *R*(0, 0.5, 0.5), *X*(0, 0.5, 0).

To further discuss the electronic properties of TE-C36 carbon, the electronic band structure and (partial) density of states ((P)DOS) are plotted in Fig. 4. As shown in the band structure, there is a direct band gap of around 2.25 eV at *Γ* point, revealing that TE-C36 carbon is a direct semiconductor. It is noted that around the Fermi level, the valence band is mainly contributed by C2 atoms, and C1 atoms contribute the most to the conduction band. Compared to graphdiyne,^19,20^ which band gap is 0.44 eV (PBE) and 1.10 eV (GW), TE-C36 carbon has a little larger band gap. Doping is one of the most effective strategies to tune the materials. For example, it is found that N-doped graphdiyne improves the Li intercalation in the lithium-ion batteries. Additionally, the band gap of TE-C36 carbon can be tuned by doping, which is very useful for future optical applications. To future discuss the modification of band gap by doping, we investigate some doped structures with the dopant replacing one or more C2 atoms, as shown in Fig. S2 (ESI†). Their band structures and density of states are plotted in Fig. S3 (ESI†). The results show that one B, N or P atom doped structures (see Fig. S2(a)–(c), ESI†), their band structures and density of states show that there are one or two band lines across the Fermi level, thus they are metallic. The dopants (B, N and P) also contribute to the Fermi level. One O atom doped structure (see Fig. S2(d), ESI†) possesses a band gap of 2.42 eV at *Γ* point (see Fig. S3(d), ESI†), thus the doping of oxygen atom enlarge the band gap of TE-C36 carbon. As shown in Fig. S3(d), ESI,† DOS is almost contributed by carbon atoms, revealing that oxygen atom only widen the band gaps but has little contribution to the conduction and valance bands. The BN co-doped structure, as shown in Fig. S2(e) (ESI†), has a band gap of 1.82 eV, revealing that the co-doping of BN decrease the band gap of TE-C36 carbon. Its DOS shows that N and B atoms contribute to the conduction and valence bands, respectively. Moreover, as shown in Fig. S2 and S3(f–h) (ESI†), the band gaps of one, two and four Si atoms doped structures are 0.96 eV, 0.36 eV and 0.08 eV, respectively, revealing that the band gap of TE-C36 carbon decreases with the increase of Si doping. Si atoms mainly contribute to the conduction bands. Therefore, the band gap of TE-C36 carbon can be tuned by different dopants.

**Fig. 5:**
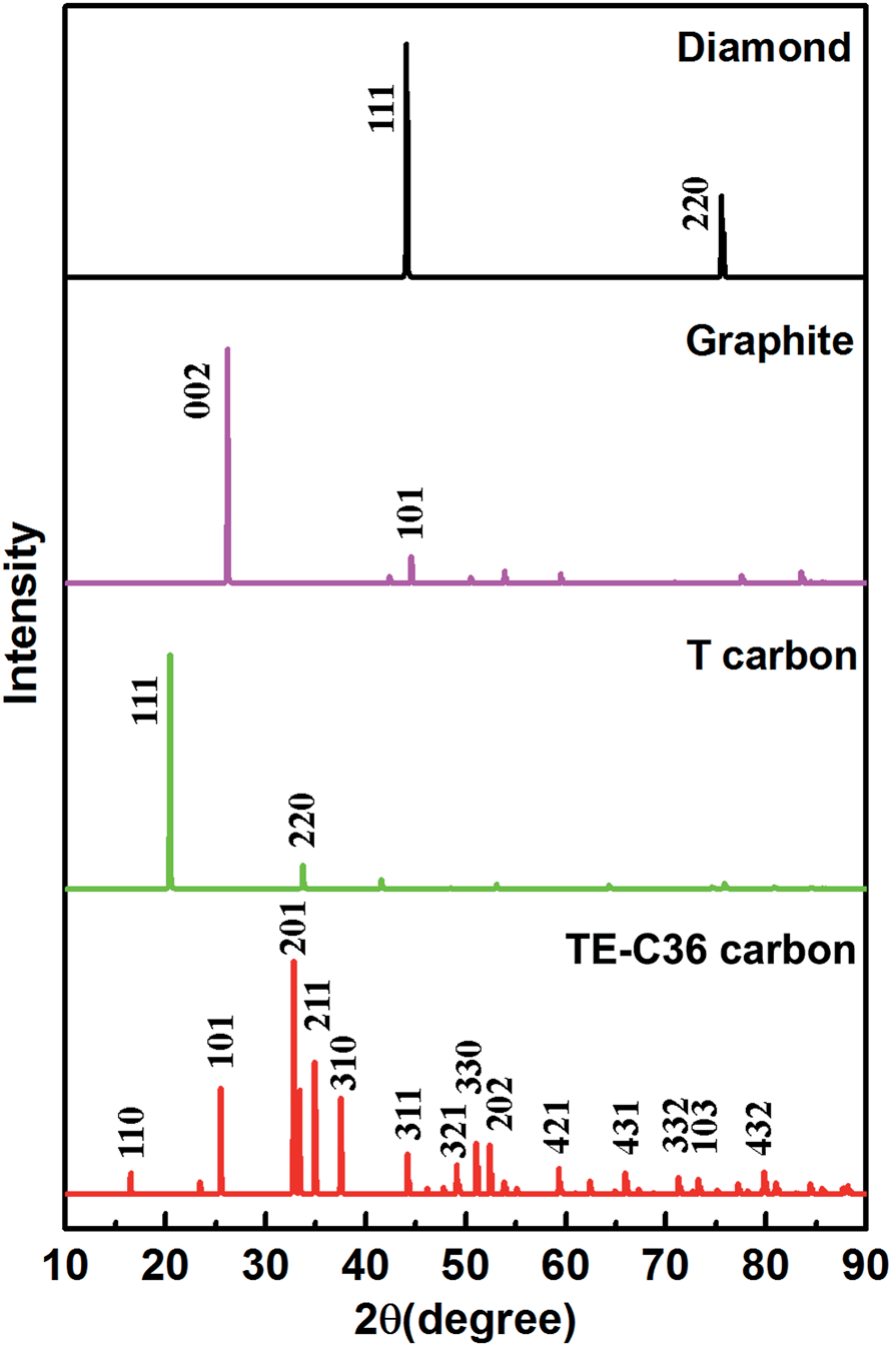
XRD of different carbon allotropes. X-ray wavelength is 1.5406 with a copper source.

Moreover, to provide more characterizations to connect the predicted results and future experiment data, the calculated X-ray diffraction (XRD) of TE-C36 carbon is plotted in Fig. 5, in comparison to diamond, graphite and T carbon. Several peaks of TE-C36 carbon are marked in Fig. 5. The first and second biggest peaks (201) and (211) locate around 33° and 35°, respectively, which are close to the peak (220) of T carbon. The third biggest peak (101) locates around 26°, which is very close to the peak (002) of graphite. The XRD features are anticipated to be experimentally measured in the future.

**Fig. 6:**
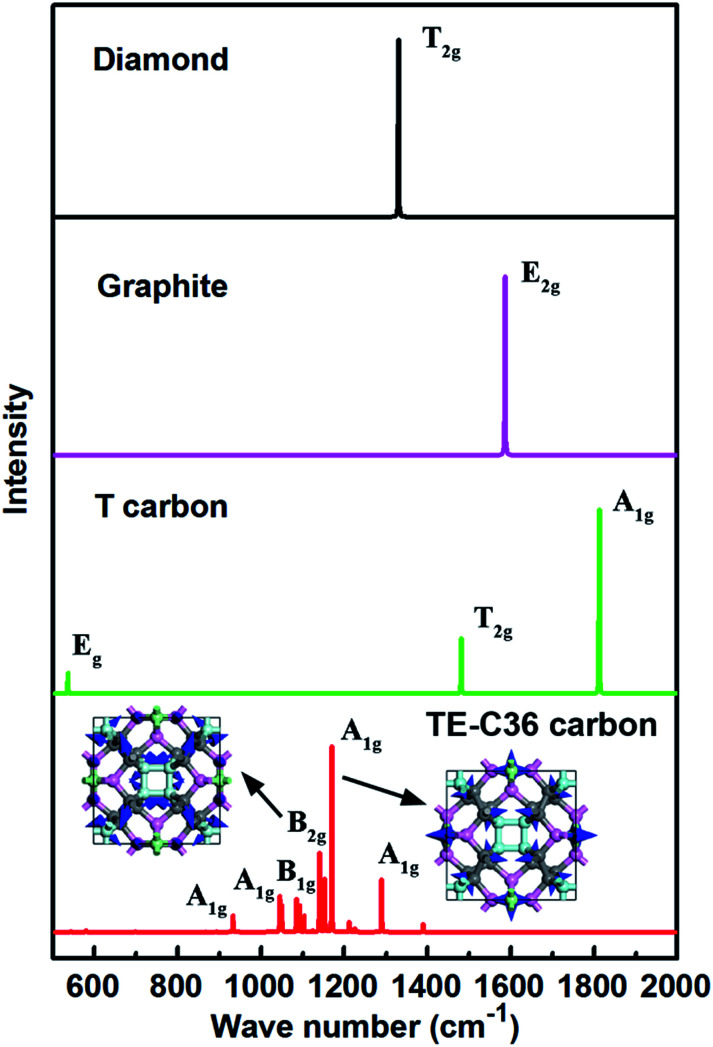
Simulated Raman spectra of different carbon allotropes. The atoms' movements of two modes are schematically marked by blue arrows in the inset.

In addition, simulated Raman spectrum of TE-C36 carbon also plotted in Fig. 6 to provide more physical quantities, compared to diamond, graphite and T carbon. The E_2g_ mode of graphite in its simulated Raman spectrum is around 1586 cm^−1^, in accordance with the experimental data.^46^ The T_2g_ mode in diamond appears around 1330 cm^−1^, in agreement with its experimental observations as well.^47^ The strongest Raman peak A_1g_ locates at 1170 cm^−1^. The second Raman peak B_2g_ appears around 1140 cm^−1^. The origin of peak 1170 cm^−1^ mainly comes from the bending of C1 and C2 atoms, and the later ones contribute the most. The peak of 1140 cm^−1^ mainly originates from the C1 atoms' bending, and the bending of C2 and C3 atoms contribute a little more than that of C4 atoms. The movements of these two modes are schematically shown in the inset. All the features can be helpful for the experimental identification of TE-C36 carbon.

During the work's preparing, we tried our best to search the possible synthesis paths from the known carbon phases (such as graphite). However the calculation results show that the energy barriers are too high (most of them are larger than 2 eV) to form the phase with squares. Considering the relative low ground state energy, we assume this structure could be synthesized from high temperature solidification (like M-carbon^13^) or laser irradiation (like T-carbon^30^) directly rather than phase transition from other carbon allotropes.

## Conclusion

In summary, we have identified a new carbon allotrope in an all-sp^3^ network in *P*4_2_/*nmc* symmetry with a mass density of 3.18 g cm^−3^. It consists of 36 atoms in a tetragonal cell. Phonon dispersion indicates that TE-C36 carbon is dynamically stable. The analysis of elastic constants implies that it is mechanically stable. Electronic band structure shows shat TE-C36 carbon is a direct semiconductor with the band gap of 2.25 eV. Simulated XRD and Raman spectra are also provided for the characterizations of future experimental observations. Due to the direct gap and a comparatively large bulk modulus, this new semiconducting carbon allotrope may possess not only potential electronic and optical applications but also mechanical application.

## Conflicts of interest

There are no conflicts of interest to declare.

## Supplementary Material

RA-008-C7RA11448F-s001
