# Supplementary material for: TE-C36 carbon: a new semiconducting phase with an all-sp3 bonding network
Source: RSC Adv. 2018 Jan 8;8(4):1846–51. doi: 10.1039/c7ra11448f (PMC9077041; doi:10.1039/c7ra11448f)
Supplement: RA-008-C7RA11448F-s001 [file RA-008-C7RA11448F-s001.pdf]

Electronic Supplementary Material (ESI) for RSC Advances.
This journal is © The Royal Society of Chemistry 2017

Supplementary Information

TE-C36 carbon: a new semiconducting phase with an all-sp^3 bonding network

Yanheng Xu [a], Yihua Lu [a], Xi Zhu*[a] and Min Wang*[b]

[a]The Chinese University of Hong Kong, Shenzhen, No.2001 Longxiang Blvd., Longgang Dist., Shenzhen 518172, Guangdong, China. Email: zhuxi@cuhk.edu.cn.

[b]Institute for Clean Energy and Advanced Materials, Faculty of Materials and Energy, Southwest University, Chongqing 400715, China. Email: minwang@swu.edu.cn.

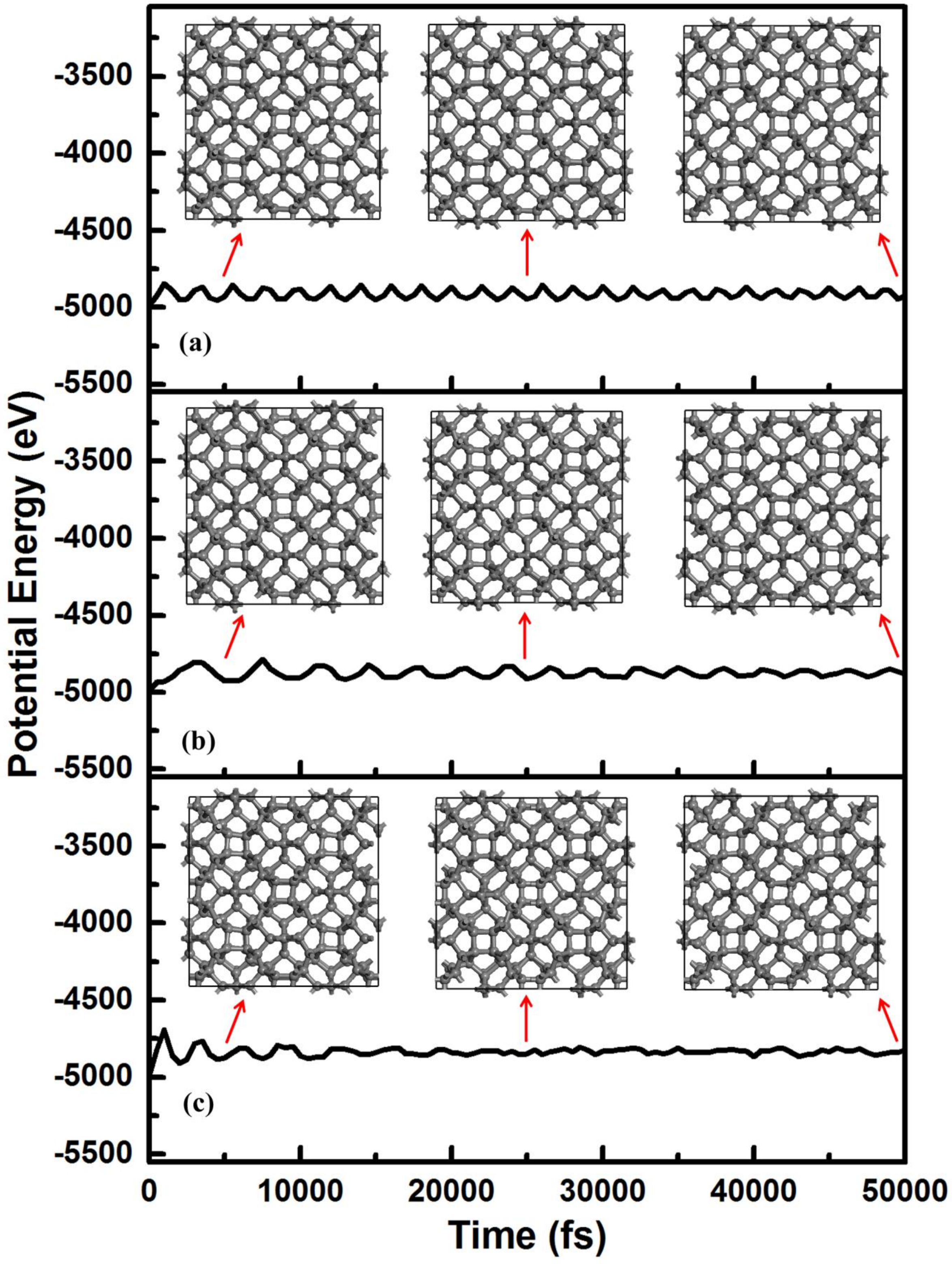

Fig. S1 Potential energy fluctuations of TE carbon in MD simulation at (a) 1000 K, (b) 1500 K and (c) 2000 K. The insets are the snapshots during the MD process.

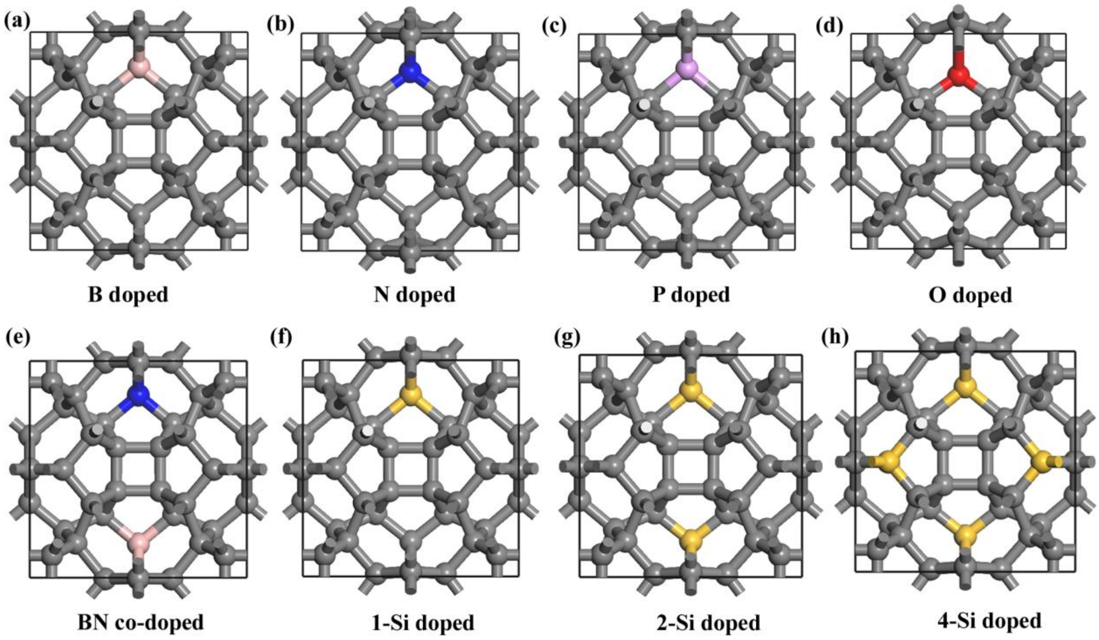

Fig. S2 (a) B doped TE carbon. (b) N doped TE carbon. (c) P doped TE carbon. (d) O doped TE carbon. (e) BN co-doped TE carbon. (f) One Si atom doped TE carbon. (g) Two Si atoms doped TE carbon. (h) Four Si atoms doped TE carbon.

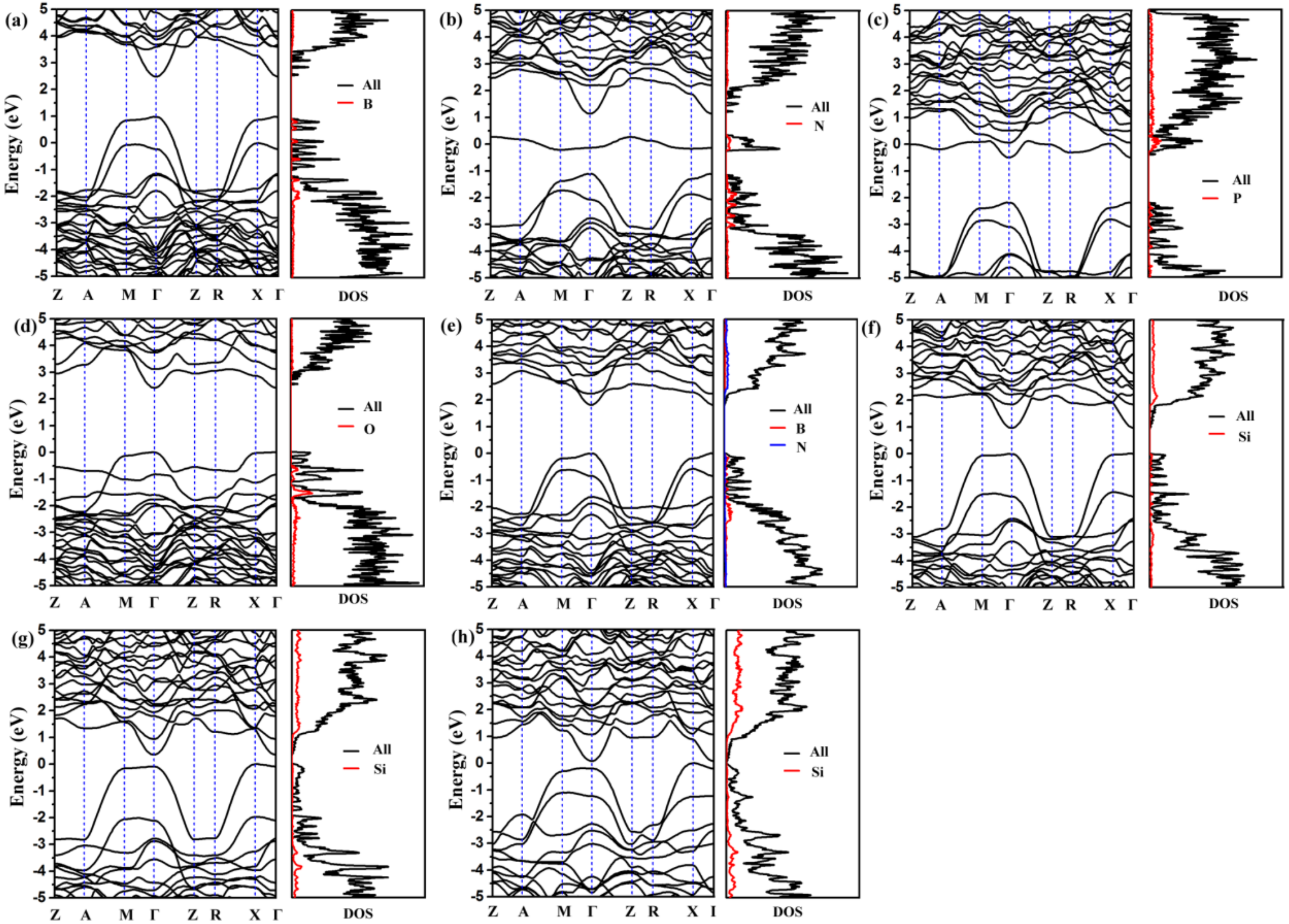

Fig. S3 (a)-(h) Band structures and partial density of states (DOS) for the doped structures in Fig. S2, respectively. Fermi level is set to zero. Z(0,0,0), A(0.5,0.5,0.5), M(0.5,0.5,0), Γ(0, 0, 0), R(0,0.5,0.5), X(0,0.5,0).
